# Diagnosis, treatment, and outcomes of hepatic artery thrombosis after liver transplantation

**DOI:** 10.1590/0100-6991e-20260010-en

**Published:** 2026-01-27

**Authors:** VIVIAN LAÍS SASAKI, JÚLIO CEZAR UILI COELHO, HENRIQUE DE AGUIAR WIEDERKEHR, MARCO AURÉLIO RAEDER DA COSTA, DIANCARLOS ANDRADE, JOÃO PAULO BARROS SANCHES, JULIO CESAR WIEDERKEHR, EDUARDO BROMMELSTROET RAMOS, ALCINDO PISSAIA

**Affiliations:** 1 - Hospital Nossa Senhora das Graças Curitiba - PR - Brasil; 2 - Hospital de Clínicas do Paraná Curitiba - PR - Brasil; 3 - Hospital Santa Isabel Curitiba - PR - Brasil

**Keywords:** Liver Transplantation, Hepatic Artery Thrombosis, Retransplantation, Transplante de Fígado, Trombose da Artéria Hepática, Retransplante

## Abstract

**Rationale:**

Hepatic Artery Thrombosis (HAT) is the most common and severe vascular complication in patients undergoing Liver Transplantation (LT), with an incidence ranging from 2% to 9% and mortality between 11% and 60%. Despite its clinical relevance, Brazilian publications on the topic remain limited.

**Objectives:**

To evaluate the diagnosis, treatment, and outcomes of HAT in patients undergoing LT at three Brazilian hospitals (Hospital Santa Isabel, Blumenau, Santa Catarina; Hospital das Clínicas, Curitiba, Paraná; and Hospital Nossa Senhora das Graças, Curitiba, Paraná).

**Methods:**

This was a longitudinal, observational, retrospective study including patients diagnosed with HAT after LT between September 1991 and December 2023 at three major Brazilian medical centers. Data were obtained from electronic medical records and institutional protocols.

**Results:**

Among 1,362 transplants performed, 35 patients developed HAT, corresponding to an incidence of 2.6%. Doppler ultrasound detected the condition within the first 48 hours in 43.8% of cases. Intraoperative assessment of arterial pulse alone was not associated with mortality (p = 0.16). Patients who underwent retransplantation showed a trend toward lower mortality (p = 0.076).

**Conclusion:**

Retransplantation is associated with reduced mortality in HAT cases and is considered the gold-standard treatment. Intraoperative Doppler ultrasound should be considered not only as a screening tool but also as an aid in surgical decision-making.

## RESUMO

**Racional:**

A trombose da artéria hepática (TAH) é a complicação vascular mais comum e grave em pacientes submetidos ao transplante hepático (TH), com incidência entre 2% e 9% e mortalidade variando de 11% a 60%. Apesar de sua relevância clínica, as publicações brasileiras sobre o tema ainda são escassas.

**Objetivos:**

Avaliar o diagnóstico, o tratamento e os desfechos da TAH em pacientes submetidos a TH em três hospitais brasileiros (Hospital Santa Isabel, Blumenau, Santa Catarina; Hospital das Clínicas, Curitiba, Paraná; e Hospital Nossa Senhora das Graças, Curitiba, Paraná).

**Métodos:**

Estudo observacional, longitudinal e retrospectivo, que incluiu pacientes com diagnóstico de TAH após transplante hepático entre setembro de 1991 e dezembro de 2023, em três centros médicos de grande porte no Brasil. Os dados foram obtidos por meio da revisão de prontuários eletrônicos e protocolos institucionais.

**Resultados:**

Entre os 1.362 transplantes realizados, 35 pacientes apresentaram TAH, correspondendo a uma incidência de 2,6%. Em 43,8% dos casos, o diagnóstico foi feito nas primeiras 48 horas por meio do Doppler. A avaliação intraoperatória do pulso arterial isoladamente não se associou à mortalidade (p = 0,16). Pacientes submetidos a retransplante apresentaram tendência à menor mortalidade (p = 0,076).

**Conclusões:**

O retransplante está relacionado à redução da mortalidade nos casos de TAH, sendo considerado o tratamento padrão-ouro. O uso do Doppler intra-operatório deve ser considerado não apenas como ferramenta de triagem, mas também como auxílio na tomada de decisões cirúrgicas.

## INTRODUCTION

Hepatic Artery Thrombosis (HAT) is a major vascular complication that may occur after Liver Transplantation (LT), with an incidence of 2% to 9% and a reported mortality rate of 11% to 60%. Despite technical advancements in LT, HAT remains one of the leading causes of graft loss, morbidity, mortality, and retransplantation^1^ ^-^ ^6^. HAT may develop immediately following anastomosis or months after transplantation and is considered early when occurring within the first 30 days post-LT^6^ ^-^ ^9^.

Its pathogenesis is multifactorial and remains unknown in some patients. Risk factors contributing to HAT include technical failures, complex anastomoses due to anatomical variations, use of vascular grafts, prolonged cold or warm ischemia times, operative time greater than seven hours, excessive arterial angulation, and small arterial lumen diameter (<3mm)^1^ ^,^ ^2^ ^,^ ^10^ ^-^ ^12^. Additional risk factors include ABO incompatibility, cytomegalovirus infection, donor age over 60 years, acute graft rejection, obesity, atherosclerosis, persistent hypovolemic shock, diabetes mellitus, and vasoconstrictor use^3^ ^,^ ^4^ ^,^ ^8^ ^,^ ^13^ ^,^ ^14^.

The transplanted liver parenchyma and its biliary structures rely exclusively on the Hepatic Artery (HA) for perfusion, as collateral vessels within the hepatic ligaments are fully severed during organ procurement. The hepatic arterial buffer response - an adenosine-mediated physiological mechanism that regulates arterial flow in response to portal venous flow - also influences hepatic perfusion. Consequently, HAT can lead to parenchymal ischemia, graft failure, and ischemic cholangiopathy^1^ ^,^ ^11^ ^-^ ^13^ ^,^ ^15^. In rare cases, patients may be asymptomatic or exhibit only transient abnormalities in liver function tests^2^ ^,^ ^11^ ^,^ ^16^.

Postoperative surveillance with Doppler Ultrasound (DU) remains the primary screening tool, enabling bedside noninvasive monitoring that can detect early reductions or absence of arterial flow and elevations in resistive indices. When findings are inconclusive or clinical suspicion persists, computed tomography angiography or conventional celiac arteriography is used to confirm arterial patency and guide therapeutic planning^5^.

Treatment options for HAT include surgical intervention, endovascular revascularization, and retransplantation¹,². The choice among these approaches depends on clinical status, timing of diagnosis, available resources, and team expertise. Surgical treatment typically consists of thrombectomy and revision of the arterial anastomosis. Endovascular options may include intra-arterial thrombolysis, thrombectomy, balloon angioplasty, and stent placement when necessary^1^ ^,^ ^13^ ^,^ ^16^.

Retransplantation remains the gold-standard therapy for HAT; however, timely access to a new graft is often limited. It is indicated when surgical or radiologic interventions fail or in cases of late HAT complicated by ischemic injury and significant hepatic dysfunction^1^ ^,^ ^8^.

Despite being associated with severe complications and unfavorable outcomes, Brazilian studies on this topic remain scarce^1^ ^,^ ^8^ ^,^ ^13^. This study aims to assess the diagnosis, treatment, and outcomes of HAT in LT patients across three major liver transplant centers in Brazil.

## METHODS

A multicenter, retrospective, longitudinal, observational study was conducted using physical and electronic medical records of patients diagnosed with HAT after LT between September 1991 and December 2023 at the following centers: Hospital das Clínicas of the Federal University of Paraná, Hospital Nossa Senhora das Graças (Curitiba), and Hospital Santa Isabel (Blumenau).

The variables analyzed included age, sex, indication for transplantation, blood type, percutaneous and surgical treatments, MELD score, intraoperative findings, transplant type (deceased donor or living donor), postoperative complications, donor characteristics, cold and warm ischemia times, operative time, retransplantation, anastomosis techniques, imaging studies, and mortality.

There were no conflicts of interest. The study was self-funded and approved by the Research Ethics Committee (CAAE: 63190022.8.0000.0096). Statistical analyses were performed using R version 4.3.3. Survival was assessed using the Kaplan-Meier method and the log-rank test. Values of p<0.05 were considered statistically significant.

### Surgical Procedure

The standard LT technique employed was either the cava-cava method (removal of the recipient’s retrohepatic vena cava) or the piggyback technique (sideto-side anastomosis between the donor’s suprahepatic vena cava and the recipient’s hepatic veins). Portal vein reconstruction was performed using a continuous end-toend anastomosis, followed by graft flushing with 500 mL of 5% dextrose.

Arterial reconstruction was performed under loupe magnification; in living donors, microsurgical techniques were used. The most common arterial anastomosis was an end-to-end reconstruction between the donor celiac trunk and the recipient common hepatic artery. Polypropylene sutures (6-0 or 7-0) were used. Routine prophylaxis with aspirin, warfarin, or other anticoagulants was not employed and was reserved only for high-risk patients.

Biliary reconstruction was most commonly performed using an end-to-end anastomosis between the bile ducts with 5-0 PDS sutures. When necessary, a Rouxen-Y hepaticojejunostomy was performed. T-tube drainage was used only until 1996.

### Postoperative Management

Doppler Ultrasound (DU) was performed on postoperative days 1 and 5, as well as in cases of clinical or laboratory suspicion. When HAT was suspected, confirmation was obtained with computed tomography angiography or arteriography. The immunosuppressive regimen included tacrolimus (or cyclosporine prior to 1998), corticosteroids, mycophenolate mofetil, and azathioprine. Liver function tests and coagulation profiles were assessed daily, and tacrolimus levels were measured twice weekly. Management of HAT was determined through multidisciplinary meetings.

## RESULTS

A total of 1,362 patients underwent Liver Transplantation (LT) across the three participating centers, of whom 35 (2.6%) were diagnosed with Hepatic Artery Thrombosis (HAT). Most patients were male (68.6%), with a mean age of 48.5 ± 17.89 years (range, 11-70 years). The most common blood type was O (n=17; 48.5%), followed by A (n=14; 40%), AB (n=3; 8.5%), and B (n=1; 2.9%). Demographic and clinical data are summarized in Tables 1 and 2.

**Table 1:** Demographic and Clinical Data of 35 Patients With Hepatic Artery Thrombosis (HAT).

	N (%)
Sex	
M	24 (68.6)
F	11 (31.4)
Hospital	
Hospital de Clinicas	14 (40)
Hospital Nossa Senhora das Graças	15 (42.9)
Hospital Santa Isabel	6 (17.1)
Blood type:	
O	17 (48.5)
A	14 (40)
AB	3 (8.5)
B	1 (2.9)
Transplant type	
Deceased donor	32 (91.43)
Living donor (intervivus)	3 (8.57)
Venous reconstruction	
Cava-cava	14 (40)
Piggyback	21 (60)
Final appearance of arterial anastomosis	
Questionable	1 (2.9)
Excellent	20 (57)
Thrombosis	3 (8.5)
NA	11 (31.5)
Anastomosis revision	
	N (%)
Yes	4 (11.4)
No	26 (74.3)
NA	5 (14.3)
Portal vein thrombosis	
No	24 (68.6)
Yes	5 (14.3)
NA	6 (17.1)
Vena cava thrombosis	
No	27 (77.1)
Yes	2 (5.7)
NA	6 (17.1)
Retransplantation	
No	17 (48.5)
Yes	18 (51.4)
Mortality	
No	21 (60)
Yes	14 (40)

**Table 2:** Liver Transplant Data for 35 Patients With HAT.

	Min	Median	Max.	Mean	SD
Age (Years)	1.66	55.16	70.75	48.27	17.89
Time of diagnosis (days)	0	5.47	27.64	7.28	8.14
ASA score	2	3	5	2.85	0.59
Actual MELD	6	11	36	13.63	7.24
Accepted MELD	19	25	40	25.11	5.14
Warm ischemia time (minutes)	30	46	85	49.46	14.02
Cold ischemia time (minutes)	50	322	780	329.3	158.6
Duration of LT (minutes)	195	340	715	367.7	110.2
Donor age (years)	13	47.5	66	40.63	17.52
Donor ICU stay (days)	1	2	6	2.69	1.4

Regarding pre-transplant clinical status, the mean ASA score was 2.8 ± 0.59, the mean actual MELD was 13.6 ± 7.2, and the mean accepted MELD was 25.1 ± 5.1. The mean warm ischemia time was 49.4 ± 14 minutes, and the mean cold ischemia time was 367.1 ± 110.2 minutes. Mean operative time for the primary transplant was 399.7 ± 140.5 minutes. Donors had a mean age of 40.6 ± 17.5 years and an average ICU stay of 2.6 ± 1.4 days. The mean time to diagnosis of HAT was 7.3 ± 8.2 days (Table 1).

Deceased-donor transplants accounted for 91.43% of cases (n=32), while living-donor transplants represented 8.57% (n=3). The piggyback technique was the most commonly used form of venous reconstruction (n=21; 60%), followed by the cava-cava method (n = 14; 40%). The arterial anastomosis was described as excellent in 20 cases (57%). A questionable arterial pulse was reported in 1 case (2.9%), and absence of flow due to immediate thrombosis was observed in 3 cases (8.5%). In 11 patients (31.4%), the intraoperative arterial flow description was not available. Anastomotic revision was necessary in 4 patients (11.4%) due to inadequate or absent arterial flow (Table 2).

Arterial anatomical variations were identified in 6 donors (17%). Four donors (11%) had a right hepatic artery originating from the superior mesenteric artery. One patient (2.8%) had a common hepatic artery arising from the left gastric artery. In another case (2.8%), reconstruction was performed using an accessory left hepatic artery anastomosed to the celiac trunk and an accessory right hepatic artery anastomosed to the gastroduodenal artery.

Two recipients required complex arterial reconstructions (5.71%) due to anatomical alterations. One had a common hepatic artery originating from a common trunk shared with the celiac trunk and the superior mesenteric artery. The other required infrarenal aortic interposition with an iliac artery graft due to atherosclerotic disease.

DU was the primary diagnostic tool and was used in 32 cases (91.4%). CT angiography was performed in 12 patients (34%) to confirm diagnosis and assess hepatic segmental viability. MR angiography was used in one case (2.85%).

Retransplantation was performed in 18 patients (51.4%) as treatment for HAT, while the remaining patients underwent reoperation with thrombectomy. Two patients underwent percutaneous arteriography attempts, both unsuccessful, followed by surgery and subsequent retransplantation (Table 2).

Post-HAT complications included biliary fistula (n=4; 12.5%), biliary anastomotic stricture (n = 3; 10.4%), and choledocholithiasis or cholangitis (n= ; 2.9%). Portal vein thrombosis occurred in 5 cases (14.3%), and inferior vena cava thrombosis in 2 cases (5.7%). Overall mortality was 40% (n=14) (Table 2).

## DISCUSSION

Hepatic Artery Thrombosis (HAT) is the most frequent and one of the most feared vascular complications after liver transplantation due to its severe clinical consequences^1^ ^,^ ^5^. 

The incidence observed in this study (2.6%) is consistent with previously reported rates^7^ ^,^ ^8^. All cases were classified as early HAT, as they occurred within the first 30 postoperative days^18^. The mean warm ischemia time in this cohort was 49.4 ± 14 minutes, reinforcing the association between prolonged warm ischemia time (≥35 minutes) and HAT risk^15^.

The most frequent arterial anatomical variation in donors was a right hepatic artery originating from the superior mesenteric artery (11%), consistent with published data. According to Herrero et al.^6^, this variation occurs in 11% to 21% of cases, being one of the most frequent anatomical alterations of the hepatic artery6. This highlights the importance of mastering appropriate surgical techniques and the careful selection of vessels for vascular reconstruction in order to reduce the risk of HAT^6^ ^,^ ^12^.

**Figure 1:**
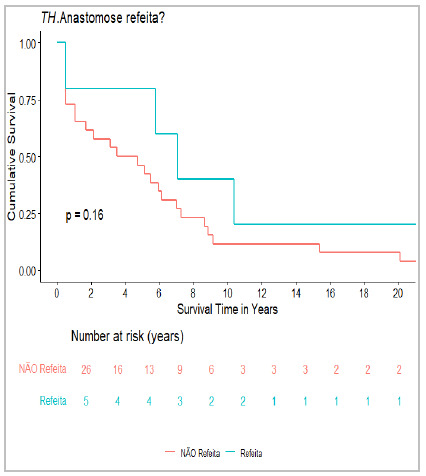
Mortality after intraoperative revision of hepatic artery anastomosis.

In our experience, HAT was diagnosed intraoperatively by evaluating the arterial pulse in three patients. The anastomosis was revised and adequate arterial flow was restored. Two patients progressed well, while one died on the second postoperative day due to refractory shock. In this study, intraoperative anastomotic revision was not associated with higher mortality (p=0.16), suggesting that more precise assessment tools may be required (Figure 1).

Previous studies^13^ ^,^ ^15^ ^,^ ^18^ ^,^ ^20^ ^,^ ^21^ highlight intraoperative Doppler ultrasound as an effective tool for assessing hepatic artery flow, identifying graft-related complications during LT, and predicting postoperative events. Moreover, because the hepatic artery has intrinsic autoregulatory mechanisms in response to changes in portal venous flow, these physiological adaptations (known as the hepatic artery tamponade response) cannot be detected by pulse palpation alone^22^. Therefore, in uncertain cases, intraoperative Doppler provides more objective assessment^15^. When unavailable, it is possible to section an artery ligated during the backtable procedure - such as the gastroduodenal artery or the splenic artery^23^ - to verify arterial flow.

The mean time to HAT diagnosis was 7.28 ± 8.14 days, similar to previously reported averages (7 days, range 1-17.5 days)^8^ ^,^ ^9^ ^,^ ^17^, underscoring the need for structured protocols for early HAT detection^9^ ^,^ ^24^. 

In this series, two patients underwent attempted endovascular recanalization, both unsuccessful, and ultimately died despite subsequent surgical revascularization or retransplantation. All patients in the cohort underwent either arterial reconstruction or retransplantation. 

**Figure 2:**
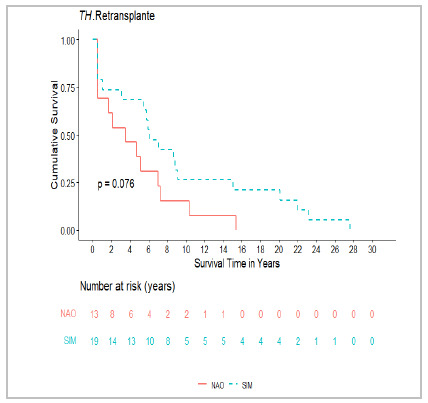
Impact of retransplantation on mortality.

**Figure 3:**
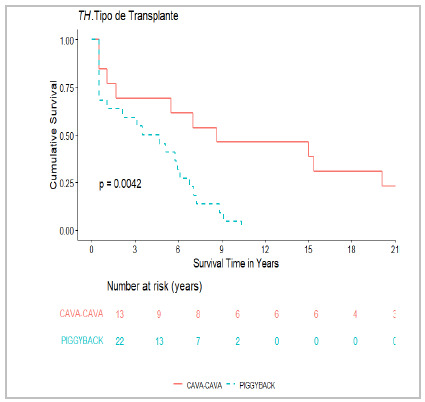
Impact of venous reconstruction on mortality.

Patients who underwent retransplantation showed a trend toward lower mortality, although without statistical significance (p=0.076), supporting its role as the gold-standard treatment for HAT (Figure 2)^8^ ^,^ ^17^. The cava-cava technique was associated with lower mortality (p=0.0042), despite literature suggesting shorter ischemia and operative times with the piggyback technique (Figure 3). This finding may reflect patient selection bias and small sample size. Mortality was the primary outcome, calculated from the diagnosis of TAH. Overall mortality in this cohort was 40%, lower than previously reported rates of 50%-60%^19^ ^,^ ^24^.

The main limitations of this study include its small sample size and retrospective design. This limitation was partially mitigated by including data from three major transplant centers. The retrospective nature was minimized by the fact that all procedures were coordinated and supervised by the same transplant team, and data were obtained from electronic medical records and institutional protocols. Moreover, given the severity of HAT, prospective trials are unfeasible.

## CONCLUSIONS

Hepatic artery thrombosis following liver transplantation is a devastating complication associated with high morbidity, mortality, graft loss, and need for retransplantation. Routine Doppler evaluation of hepatic vessels after LT is essential for early diagnosis. Although intraoperative anastomotic revision did not demonstrate statistical significance, additional vascular assessment methods may be indicated in uncertain cases. Retransplantation remains the suggested gold-standard treatment for HAT.
